# Water Tree Propagation in a Wide Temperature Range: Insight into the Role of Mechanical Behaviors of Crosslinked Polyethylene (XLPE) Material

**DOI:** 10.3390/polym13010040

**Published:** 2020-12-24

**Authors:** Siyan Lin, Kai Zhou, Yuan Li, Pengfei Meng

**Affiliations:** School of Electrical Engineering and Information, Sichuan University, Chengdu 610065, China; LIN_041@126.com (S.L.); hvliyuan@scu.edu.cn (Y.L.); mpf@scu.edu.cn (P.M.)

**Keywords:** power cables, XLPE insulation, temperature, water trees, mechanical behaviors, thermal motion

## Abstract

To understand the propagation characteristics of water trees at a wide temperature range, this paper presents the effect of mechanical behaviors on the sizes of water trees. An accelerated water tree aging experiment was performed at −15 °C, 0 °C, 20 °C, 40 °C, 60 °C, and 80 °C for crosslinked polyethylene (XLPE) specimens, respectively. Depending on the micro observations of water tree slices, water tree length is not always increasing with the increase in temperature. From 0 °C to 60 °C, water tree length shows a trend from decline to rise. Above 60 °C, water tree length continues to reduce. Dynamic mechanical analysis (DMA) shows that the glass transition temperature of the new XLPE specimen is about −5 °C, and the α-relaxation is significant at about 60 °C. With the increase in temperature, the XLPE material presents different deformation. Meanwhile, according to the result of the yield strength of XLPE at different temperatures, with the increase in temperature, the yield strength decreases from 120 MPa to 75 MPa, which can promote the water tree propagation. According to the early stage in the water tree propagation, a water tree model was constructed with water tree branches like a string of pearls to calculate electric field force. According to the results of electric field force at different expansion conditions, with the increase in temperature, due to expansion of the water tree branches, the electric field force at water tree tips drops, which can suppress the water tree propagation. Regardless of high temperature or low temperature, the water tree propagation is closely related to the mechanical behaviors of the material. With the increase in temperature, the increased deformation will suppress the water tree propagation, whereas the decreased yield strength will promote water tree propagation. For this reason, at different temperatures, the promotion or suppression in water tree propagation is determined by who plays a dominant role.

## 1. Introduction

Water trees are regarded as one of the principal aging factors of crosslinked polyethylene (XLPE) cables [1,2,3]. Generally, cables buried in soil would withstand prolonged temperature gradients during their service life. These temperature variations and electrical stresses can cause breakdown in XLPE cables [4]. The chemical and physical structures of XLPE and other insulation systems of power cables can be altered at varying degrees, which can cause different propagation characteristics of electrical trees and water trees [5,6]. Electrical and thermal aging for polymeric and nanocomposite- based polymeric insulating material [7,8,9,10,11], and also the aging mechanism of in-service cables due to water trees and electrical trees at different temperatures, has become a topic of interest in recent years [12,13,14,15].

Many publications have linked breakdown phenomenon in polymeric material to space charge accumulation and trap levels at different temperatures and electric field [16,17,18,19,20]. However, very few contributions have presented understanding towards water tree propagation at different temperatures, and very few possible mechanisms have been proposed [21,22,23]. However, water tree propagation usually changes with applied aging temperatures, resulting in contradictory conclusions regarding applying different aging temperature ranges. Some studies have found that water trees propagate more easily at low temperatures [13,14], whereas others indicate that water trees propagate more easily at high temperatures [22]. A recent work [23] even finds that there are transition temperatures for water tree propagation with the rise in temperature. However, there still exists no unified physical model to explain the high initiation rates at lower temperatures and the minimum initiation rates at high temperatures (from 40 °C to 60 °C). In fact, many researchers agree that water tree propagation characteristics are closely associated with the molecular thermal motion of polymer [21,22,23,24,25], which strongly depends on temperature. The thermal motion of molecules affects the mechanical behaviors of material (e.g., deformation and yield strength), which can further influence water tree propagation characteristics. However, the influence of chain thermal motion on water tree propagation, especially in a wide temperature range, is not thoroughly understood. Therefore, it is desirable to establish a physical model to understand the relationship between water tree propagation characteristics and molecular thermal motion at different temperatures.

To gain insight into the relationship between chain motion and water tree propagation, water tree propagation at different temperatures was investigated in this paper. In addition, mechanical behaviors in the XLPE material at different temperatures were measured using dynamic mechanical analysis (DMA) and tensile test.

## 2. Experimental Setup

### 2.1. Specimen Preparation

The low density polyethylene (LDPE) particles weighing 34.5 g, 2% *w*/*w* dicumyl peroxide (DCP), and other additives were first blended and placed in the mold, which was then preheated in the hydraulic machine (manufactured by Beijing Future Material Technology Co., Ltd., China) at a temperature of 115 °C for 10 min. After the treatment, the blends were crosslinked for 15 min at 170 °C and 15 MPa. The mold is square-shaped with a size of 100 × 100 × 3 mm^3^. The prepared XLPE materials were then cut into 50 × 50 mm^2^ square-shaped specimens to be subjected to different temperature accelerated aging experiments.

**Figure 1:**
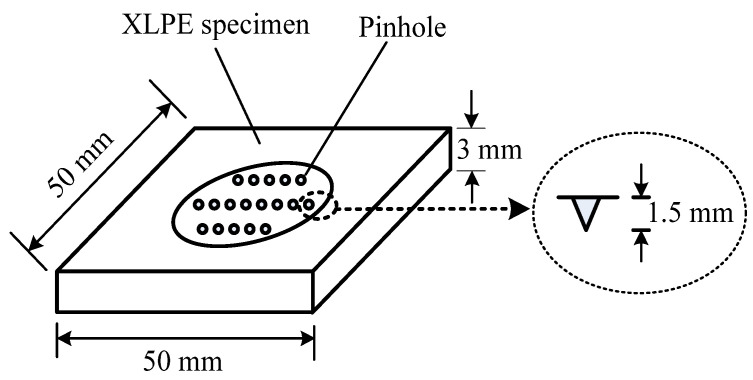
Configuration of the XPLE (crosslinked polyethylene) sheet specimen.

Figure 1 shows the configuration of the XLPE specimen. The average sheet thickness is 3 mm, with a 25-mm diameter circular area chosen as the active aging zone. Eighteen pinholes with depths of 1.5 mm were produced in the aging area, in advance, by the metal needle [6,13]. The needle has a tip radius of 4.0 ± 0.5 μm. All specimens were prepared at 20 °C. 

### 2.2. Water Tree Aging Experimental Setup and Optical Observation

**Figure 2:**
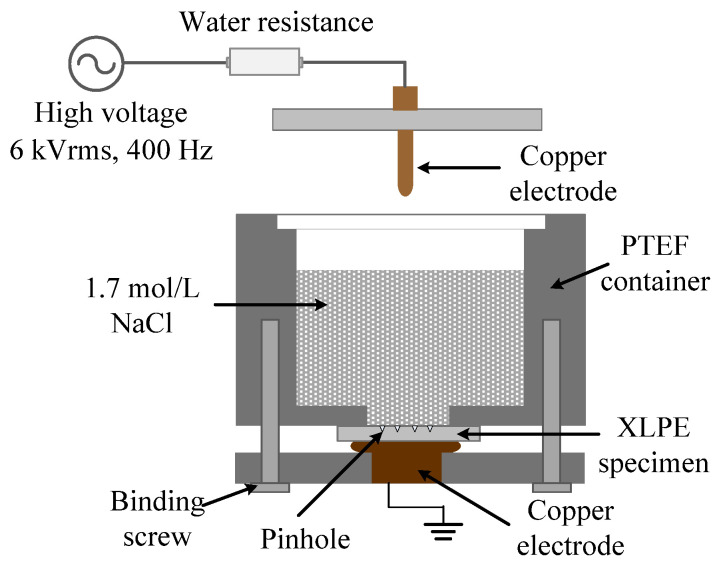
Water tree accelerated aging experimental setup [8].

Figure 2 shows a schematic of the accelerated water tree aging experimental setup recommended by IEC/TS 61956. NaCl solution was used at a concentration of 1.7 mol/L. A 6 kV rms voltage with a frequency of 400 Hz was applied to the NaCl solution through an upper copper electrode in contact with the solution. The XLPE specimen was fixed on the bottom of the container to ensure the aging area was completely immersed in the solution. The bottom copper electrode was grounded. A controlled oven and a controlled freezer (both are manufactured by Beijing Surui electronic equipment Co., Ltd, Beijing, China) were used for water tree aging at different temperatures. Four specimens of each group were exposed to isothermal environments at −15 °C, 0 °C, 20 °C, 40 °C, 60 °C, and 80 °C, respectively.

After 22 days of aging, water tree aged specimens at different temperatures were respectively sliced around the pinholes by a microtome manufactured by Jinhua Yidi equipment Co., Ltd, Jinhua, China. The minimum resolution for the slices is 1 μm, and the slicing accuracy is ±5%. The thickness of the slice is about 100 μm, and the slices were dyed in a 90 °C methylene blue solution for 30 min. After the treatment, the slices were observed by an optical microscope (manufactured by Guangzhou Ming-Mei Technology Co., Ltd, Guangzhou, China) under different magnifications.

### 2.3. Dynamic Mechanical Analysis

A dynamic mechanical analysis (DMA) device was used to measure the mechanical properties of the material with cyclic stress under different temperatures. The DMA is an accurate method that can reflect the relaxation of molecular chains as a function of temperature. To investigate the mechanical behaviors of XLPE material at different temperatures, DMA device Q800 (manufactured by TA Instrument Waters-LLC, New Castle, DE, USA) was employed to characterize and interpret the mechanical properties of the new XLPE specimens. The specimens were heated at a ramp rate of 2 °C/min under nitrogen atmosphere, over a heating range of −130 °C to 90 °C, and at a frequency of 1 Hz.

### 2.4. Tensile Test

**Figure 3:**
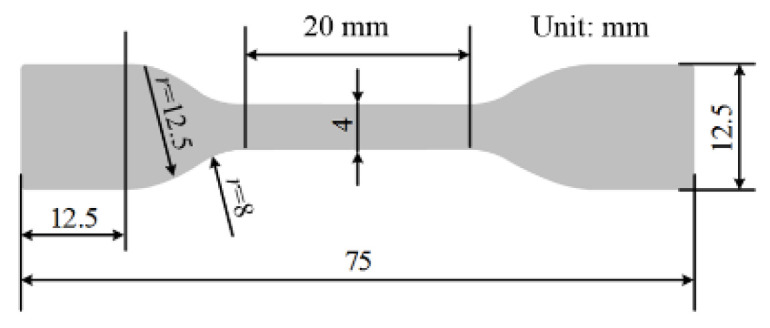
Configuration of XPLE dumbbell-shaped specimen for tensile test.

To investigate the mechanical strength of the material, according to the method in a standard (GB/T 1040-2006, tensile strength test of plastics), a tensile test was also performed. The tensile testing machine JDL-5000N (manufactured by Ji’nan Chuanbai equipment Co., Ltd, Ji’nan, China) was used in the test. Twenty-five dumbbell-shaped specimens with 75 mm in length were produced for the tensile test, with an average thickness of (1 ± 0.2) mm. Figure 3 shows the configuration of each dumbbell-shaped specimen. Five specimens from each group were exposed to isothermal environments at 0 °C, 20 °C, 40 °C, 60 °C, and 80 °C, respectively. The tension was always applied to the specimens until the break of specimens with a stretching rate of 250 mm/min.

## 3. Experimental Results

### 3.1. Observations of Water Trees

**Figure 4:**
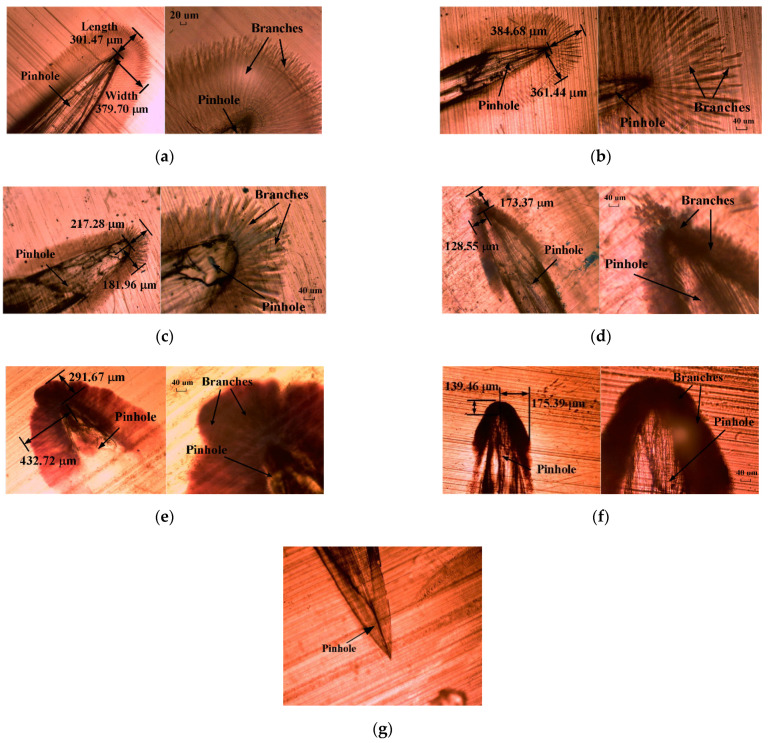
Water tree morphologies at different temperatures after 22 days of aging (24 h per day): (**a**) −15 °C; (**b**) 0 °C; (**c**) 20 °C; (**d**) 40 °C; (**e**) 60 °C; (**f**) 80 °C (×64 and ×160 magnifications); (**g**) pinhole without water tree aging.

Figure 4 shows the morphologies of water trees at six different temperatures. The slices were observed with magnifications of 64 times and 160 times, respectively. It is observed that the water trees are radial structures with branches. They are always initiated from the tips and the sides of the pinholes.

As shown in Figure 4, the morphologies of water trees significantly change with the applied aging temperatures. Below 40 °C, the individual branches can be observed. With the rise in temperature, the branches tend to be wider, from several microns to tens of microns. At the higher temperature ranges (above 40 °C), the water tree area is found to be dark and no individual branches can be clearly seen. As all the slices were dyed in the same conditions, it can be concluded that water tree branches tend to become wider and denser as the temperature increases [24]. With the increase in temperature, these water tree branches become wider, and some water tree branches even overlap together. For this reason, individual branches cannot be observed. The phenomenon can be attributed to the changes of microstructure in XLPE material.

**Figure 5:**
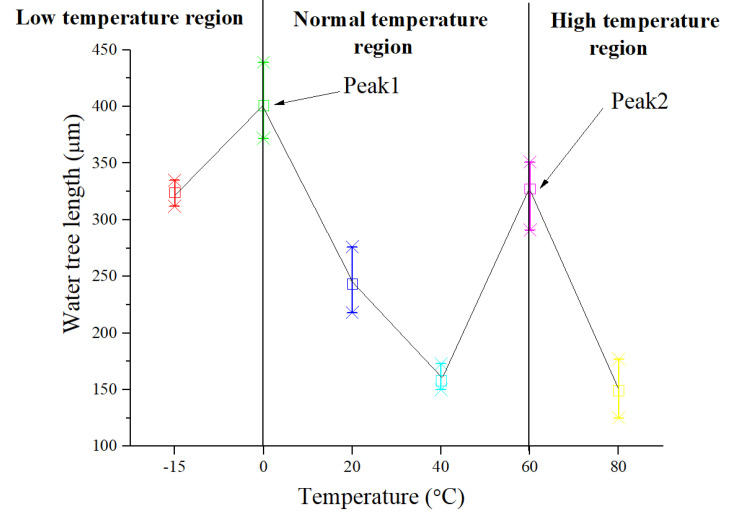
Maximum water tree length at different temperatures.

To further understand how water trees propagate as temperature rises, the average length and width for five water trees were counted for every temperature. The measurement method in length and width of water trees is shown in Figure 4a. Due to the changing trend with temperatures in width being similar to the trend in the length of the water tree, just the results of length are shown by a statistical chart in Figure 5.

According to the results in Figure 5, the length of the water tree does not always increase or decrease with the rise in temperature, and it presents nonlinear changes. Due to the changes being possibly related to the mechanical properties of the material which are being affected by the molecular thermal motion at different temperatures, in the next section, the relationship between them will be discussed in detail. The trend curve of water tree length in Figure 5 was separated into three regions: low-temperature region (below 0 °C), normal temperature region (0–60 °C), and high-temperature region (above 60 °C). In each region, the water tree length exhibits different trends. Below 0 °C, with the decrease in temperature, water tree length decreases. In the normal temperature region, water tree length presents a trend in the form of firstly increase and then decrease. Above 60 °C, water tree length reduces with the rise in temperature. There are two peaks of water tree length at 0 °C (Peak 1) and at 60 °C (Peak 2). This implies a transition temperature around 0 °C and 60 °C, at which the water tree propagation mechanism can change. As a result, in the next section, water tree propagation behaviors in the three temperature regions were discussed, as shown in Figure 5.

### 3.2. Results of Dynamic Mechanical Analysis

According to the knowledge of physics of polymer [26], the elastic modulus E* is defined by the Equation (1):

E* = *E*’ + i*E″ (1)

where *E′* represents the real part of the elastic modulus or is called storage modulus, which means the stored energy of material subjected to elastic deformation; *E″* is the imaginary part of the elastic modulus or is called loss modulus, which represents the energy loss using thermal energy during deformation. 

**Figure 6:**
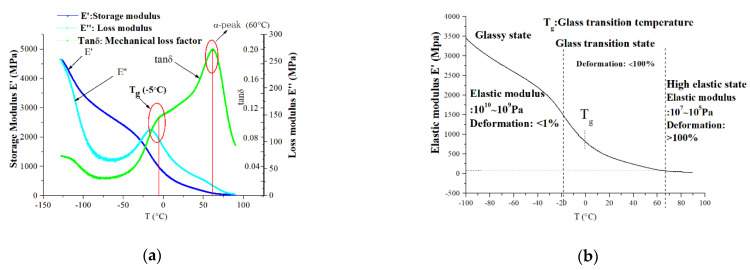
DMA (dynamic mechanical analysis) spectrum of new XLPE specimen: (**a**) DMA spectrum of new XLPE specimen; (**b**) Mechanical states at different temperatures.

Similarly, the mechanical loss factor is Tanδ = *E″*/*E′*. Figure 6a shows typical curves of the new XLPE material obtained by the DMA device. At the glass transition temperature, the storage modulus *E′* and the mechanical loss factor tanδ will present a peak at the same time, and the loss modulus *E″* will have the faster rate of descending. For this reason, according to the three curves in Figure 6a, the glass transition temperature (Tg) can be determined to be around −5 °C. In fact, the curve of tanδ has two peaks, and the peak of Tg does not look obvious because of the superposition of two peaks in the curve. In general, even though pure PE’s glass transition temperature is about −70 °C, the XLPE greatly increases the glass transition temperature because of its crosslink effect. For this reason, XLPE’s glass transition temperature is greatly higher than that of PE. 

According to the typical curves of the new XLPE material in Figure 5, it can be observed that a typical relaxation process appears at about −5 °C, which is usually associated with the glass transition of XLPE material [26]. The relaxation means that once the temperature is higher than −5 °C, chain segment motion becomes active. Moreover, at about 60 °C, the DMA spectrum shows a significant relaxation process, which peaks at 60 °C. It is generally accepted that the relaxation appearing around 60 °C can arise from the mobile units in the crystalline phase of PE in the form of chain twists, usually called α-relaxation [21,26]. The two important relaxation processes—the motion of the segment and the motion of the crystalline phase—can lead to the changes in modulus and water tree propagation behaviors.

Combining the results of DMA with the observation results of water trees in Figure 4, the glass transition temperature of the new specimen is about −5 °C, and the α-relaxation is significant at about 60 °C. The two temperatures are almost consistent with the temperatures corresponding to the peaks of water tree length in Figure 4. The relationship provides important evidence that the mechanical behaviors of material are closely related to the propagation of water trees.

According to the different modulus at different temperatures, three different mechanical state regions can be approximately divided in Figure 6b, which are glassy state, glass transition state, and high elastic state, respectively [26]. At different mechanical states, water tree propagation will present different characteristics. According to the three different temperature regions in Figure 5, the low-temperature region (below 0 °C) is nearly at the range of the glassy state. Under the glassy state, the material has less deformation (<1%), which is caused by the change of bond distance and bond angle. The normal temperature region (0–60 °C) is at the range of the glass transition state. During the temperature range, chain sections begin to move, and the deformation further increases, which is usually less than 100%. The high-temperature region (above 60 °C) is approaching the range of the high elastic state. Due to motion in the crystalline phase of PE starting, the deformation will greatly increase and is more than 100%. As a result, water tree sizes at different temperature regions as shown in Figure 5 can be mainly dominated by the mechanical properties of the material at different temperatures as shown in Figure 6b. In the next section, the relationship between them will be discussed in detail.

### 3.3. Results of Yield Strength at Different Temperatures

**Figure 7:**
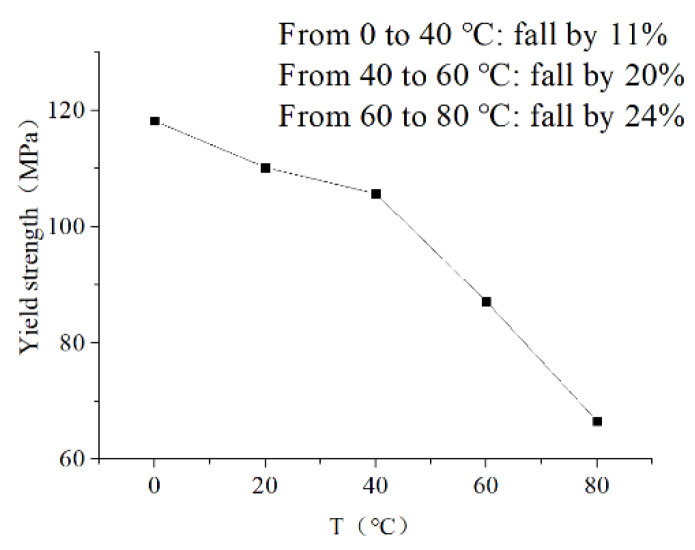
Yield strength of XLPE specimens at different temperatures.

According to the described method in Section 2.4, the yield strength of XLPE specimens is shown at different temperatures as shown in Figure 7. Depending on the results, with the increase in temperature, the yield strength of XLPE material decreases. From 0 °C to 40 °C, the yield strength has a slight reduction, which just falls by 11%. However, above 40 °C, it sharply descends. From 40–60 °C, it descends by 20%, and it continues to descend by 24% from 60–80 °C. According to the electro-mechanical mechanism of water tree propagation, the reduction of yield strength will promote the propagation of water trees [27]. As a result, the water tree propagation will be promoted with the increase in temperature. However, water tree length reduces in the normal region (0–60 °C) with the increase in temperature. For this reason, the reduction of yield strength is just one side of the effect of temperature on water tree propagation. On the other side, the rise in temperature can affect the deformation of the material, which also can influence water tree propagation. 

From Figure 5, in the low-temperature region (below Tg), the water tree length decreases with the decrease in temperature. When the material is in a glassy state, the movement of chain segments is frozen, and the deformation of the material is very small and less than 1%. The scission of chain segments is not easy to occur because of the difficult movement of chain sections. For this reason, the water tree propagation can be suppressed with the decrease in temperature. In addition, the diffusion of water molecules and ions is also inhibited when the temperature is quite low. Therefore, below Tg, with the reduction in temperature, the water tree propagation rates will slow down. However, because the deformation of a material is very low (less than 1%) in the low-temperature region, which leads to less size of voids and channels in water tree branches, the electric field force in the low-temperature region is stronger than that in the normal temperature region at water tree tips. As a result, as seen from Figure 5, the water tree length in the low-temperature region can be longer than that in the normal temperature region. Due to less deformation of the material, it is also difficult to diffuse the water molecules into the sides of water tree branches, therefore, the water tree branches also should be thinner than that in the normal temperature region. For this reason, as seen from Figure 4a,b, the water tree branches are thin and easy to distinguish in the temperature region.

With the rise from 0 °C to 60 °C in temperature, due to the reduction of elastic modulus (reduced by 70%), based on Hooke’s law, the size of voids and channels should increase with the rise in temperature. As a result, as seen from Figure 4c,d, it can be observed that the water tree branches become wider in the temperature range than those in the low-temperature region. Generally, XLPE material is a semi-crystalline polymer. According to the results of X-ray Diffraction (XRD), the crystallinity of a new sample is 49% at room temperature. Depending on the results of DMA as seen in Figure 6, from 0 °C to 60 °C, the chain segment motion causes the deformation of XLPE material, and the crystalline phase still does not move at the same time. For this reason, under the effect of electric field force, the material’s deformation should be limited by the crystalline region, which should not exceed the volume of the amorphous region. Moreover, the free volume of PE is about 6–8% at 22 °C [27], which generally increases with a rise in temperature. Due to the deformation degree being usually related to the free volume in material, under such limited free volume, the XLPE material’s deformation should be limited, which can be less than 100% from 0 °C to 60 °C. Compared with the water tree morphology at 60 °C, the water tree branches in the temperature range still can be recognized, as seen in Figure 4c,d, and its color is lighter than that of water tree at 60 °C. This is due to the diffusion of water molecules in the water tree region being restricted in the limited volume by the crystalline region, and sizes of voids and channels in water tree branches being limited with the rise in temperature in the temperature range. For this reason, water tree branches can be recognized, and they do not completely overlap each other in the normal temperature region.

## 4. Discussion

### 4.1. Water Tree Propagation Based on Electro-Mechanical Fatigue

According to the water tree propagation mechanisms [1,23], the mechanical deformations of material can play an important role in the propagation of water trees under Maxwell stress. Once being placed in an inhomogeneous electric field, water molecules and ions will move toward the spots with higher electric fields and cause the permittivity and conductivity to change. Under the AC electric field, Maxwell stress would occur and cyclic stress will apply to the material [27]. The stress *F* can be defined in Equation (2):

(2) $$F=\left(\varepsilon_{0}/2\right)\nabla \left(\varepsilon_{r}-1\right)E^{2}$$

where *ε*_0_ represents the permittivity of vacuum; *ε_r_* represents the relative permittivity of dielectric; *E* represents the electric field strength. The effect of cyclic fatigue can cause chain scission under the effect of Maxwell stress. With the increase in aging time, the chain scission will lead to the formation of microcracks or microvoids and further result in the propagation of water trees [1,2].

According to Equation (2) [27], the condition of water tree propagation is defined in Equation (3):

(3) $$\frac{1}{2}N\varepsilon_{r}\varepsilon_{0}nv_{0}E^{2}\geq Y\times V$$

where *n* is the number of cavity-filled water; *N* is the number of periods of an electric field; *v*_0_ is the volume of the cavity-filled water; *E* is electric field strength; *Y* is the yield strength of the material; *V* is water tree volume. 

For a given cavity filled with water, the cavity will grow when the electric field force exerted on the material is larger than the yield strength of the material after *N* cycles. Actually, $1/2\varepsilon_{r}\varepsilon_{0}E^{2}$ represents the electric field energy density in the given cavity-filled water, which also represents the electric field force per unit area or pressure [28,29]. When the accumulated electric field force in the cavity filled with water is more than the yield strength *Y*, the cavity will grow and the water tree will propagate with it. As a whole, the water tree propagation depends on the mutual effect between the electric field force on the cavity filled with water and the mechanical properties of the material (e.g., yield strength *Y*). When the electric field force on the cavity filled with water increases, or yield strength *Y* decreases, the water tree will rapidly propagate [30,31].

The results and how they can be interpreted from the perspective of previous studies and the working hypotheses should be discussed. The findings and their implications should be discussed in the broadest context possible. Future research directions may also be highlighted.

### 4.2. Water Tree Model and Electric Field Distribution

**Figure 8:**
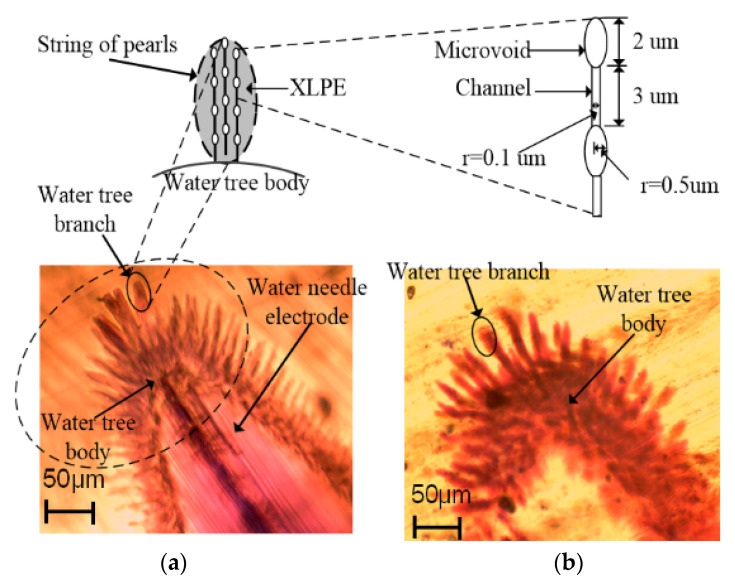
Water tree morphologies after one week and two weeks of water tree accelerated experiments at 20 °C: (**a**) After one week; (**b**) After two weeks.

As shown in Figure 4, these water trees experienced the water tree accelerated experiment of 28 days at different temperatures. To understand the characteristics of water trees at different temperatures, the early period of the water tree growth needs to be known, then an electric field simulation model will be constructed to analyze them. As a result, based on the water tree aging experimental setup described in Section 2.2, a water tree is shown in Figure 8a after the aging experiment of one week at 20 °C. The water tree includes a water tree body and some forked water tree branches, and the branches are growing on the water tree body. The length of water tree branches is very short, and its length is about 30 μm and its width is 10 μm. These branches are very sparse and are easy to recognize. From Figure 8b, after two weeks, these branches become wider and longer than those after the aging experiment of one week, but its morphology is still similar to that of a water tree after the aging experiment of one week. 

Based on the water tree morphologies in the early period observed in Figure 8, a water tree includes a water tree body and some forked branches. Generally, water tree branches consist of water-filled voids and channels to form the “string of pearls” model [27]. The water-filled microvoids can be several microns in radius, and the channels are several to tens of microns in length and tens of nanometers in width. Due to the width of branches, as shown in Figure 8a, reaching about 10 micrometers, the larger water tree branches can include a few small branches [32]. Each small water tree branch includes the elliptic voids with a major axis of 2 μm and a minor axis of 1 μm, and the channels with 3 μm in length and 100 nanometers in width [32].

**Figure 9:**
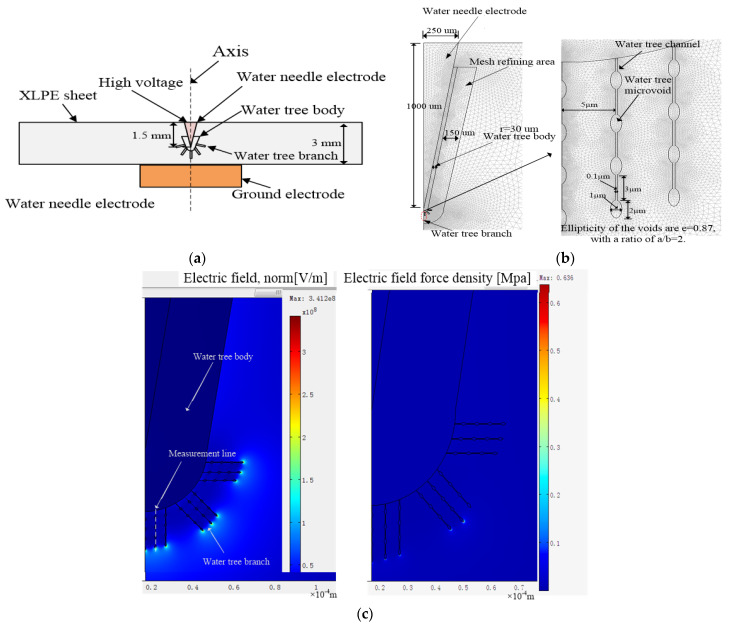
The water tree model in a sheet specimen for electric field calculation: (**a**) Specimen and water tree model; (**b**) Water tree main body, water tree channel, and water-filled voids; (**c**) Electric field distribution and electric field force density of water tree.

To simulate the electric field distribution of water tree tips as shown in Figure 8, we assume that there are five larger branches around the main body of the water tree, and each larger branch consists of a few small branches with the water-filled voids and the channels. The experimental specimen and the water tree model is shown in Figure 9a. The needle is vertically inserted into the sheet at a depth of 1.5 mm. A voltage of 6 kV rms with a frequency of 400 Hz has been applied to the upper surface of the XLPE sheet, and the bottom copper electrode is grounded. According to the model, an axial symmetry simulation model was constructed, as shown in Figure 9b, by using the finite element analysis software COMSOL Multiphysics, and a mesh refining area was added to refine the mesh [33,34,35]. Some geometric parameters are set in the model, and the finite element mesh consists of 311,680 triangle elements. The relative permittivity of XLPE is set to 2.3, and the conductivity is 1 × 10^−17^ S/m. According to the parameters in [33], the relative permittivity in the water tree region is set to 10 and the conductivity is 1 × 10^−8^ S/m. 

According to the model in Figure 9b, the electric field distribution can be calculated, as shown in Figure 9c. According to the relationship between electric field energy and force, the electric field force density is equal to $1/2\varepsilon_{r}\varepsilon_{0}E^{2}$, where *E* represents electric field strength. The electric field force density is also shown in Figure 9c. At the tips of water tree branches, the electric field force is very strong. In the next section, the effect of temperature on the electric field force will be discussed in detail.

### 4.3. Water Tree Propagation at Different Temperatures

**Figure 10:**
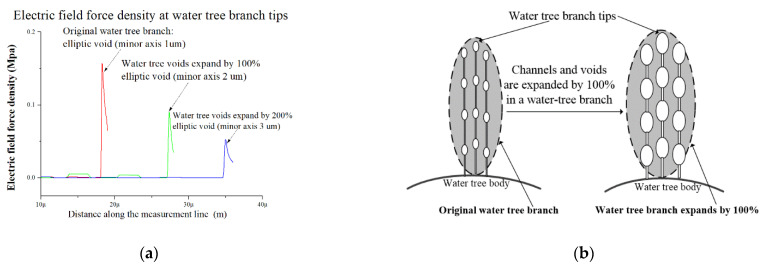
Electric field force density and expansion of water tree branches at higher temperature: (**a**) Electric field force density at water tree branch tips; (**b**) Expansion of water tree branches at a higher temperature.

For a given void or a channel in a water tree as shown in Figure 8, due to the decline of elastic modulus with the rise in temperature, the XLPE material will become softer, which will result in easier diffusion of water molecules driven by the electric field force. Based on Hooke’s law, the size of voids and channels in the water tree both should expand due to the reduction of modulus. Moreover, with the rise in temperature, the water molecules easily diffuse around voids and channels because of increased molecules’ thermal motion. As a result, from Figure 4, it can be observed that the branches in water trees become wider with the increase in temperature. To simulate the water tree conditions under different temperatures, we assume the size of voids and channels in the water tree increases with the rise in temperature. According to the model and parameters in Figure 9b, electric field force densities at the void tips are shown at different expansion times of water tree branches as shown in Figure 10a. With the expansion in voids’ size, the electric field force obviously decreases at water tree tips. As shown in Figure 10b, because of the augment in size of voids with the increase in temperature, the radius of curvature at the void tip enlarges with it, and electric field force decreases at the void tip. As a result, it is possible that water tree propagation will be inhibited with the rise in temperature. 

When XLPE is in the normal temperature (0–60 °C), according to the result in Figure 6b, maximum deformation increases by 100% at the range of 0 °C to 60 °C. For this reason, we assume the size of the voids and channels will expand by 100%. From Figure 10a, compared with the electric field force of the original water tree, the electric field force at the water tree tip reduces by 42% with the expansion of 100% in void’s size. Meanwhile, the yield strength of XLPE just decreases by 11% from 0 °C to 40 °C. According to Equation (3), the electric field force exerted on material and yield strength of material together determines the water tree propagation. The decrease of electric field force will inhibit the propagation of water trees, whereas the decrease of yield strength will promote the propagation of water trees. 

From 0 °C to 40 °C, because the effect of reduction of electric field force can be more than that of reduction of yield strength, the water tree propagation will be suppressed. However, with the further increase in temperature from 40 °C to 60 °C, because the free volume can limit the further deformation, the deformation barely continues to increase. The electric field force at water tree tips no longer decreases with the further increase in temperature. However, according to the result in Figure 7, the yield strength reduces by 20% from 40 °C to 60 °C. As a result, from 40 °C to 60 °C, with the increase in temperature, because the effect of reduction of yield strength is more than that of reduction of electric field force on the water tree propagation, the growth of water tree can be promoted.

From 60 °C to 80 °C, according to results in Figure 6, the motion of the crystalline phase of PE starts to be active, so the deformation will greatly increase, and can be much more than 100%. The material’s mechanical properties approach a high elastic state. Due to the motion of the crystalline phase, water molecules diffuse into the larger volume without the limitation of the crystalline phase, and water tree branches can further expand and overlap each other in the temperature range. For this reason, as seen from Figure 4e,f, individual branches cannot be observed, and the color of the water tree becomes darker than that of the water tree in the normal region. Under this situation, we assume the channels and voids in the water tree expand by 200%. As shown in Figure 10a, it can be observed that the electric field force declines because of the further expansion of water tree branches. Compared with the electric field force with the expansion of 100%, the electric force drops by 43% with the expansion of 200%. For this reason, above 60 °C, due to the movement of the crystalline phase, the material will present a high elastic state, and the deformation will greatly increase, so the electric field force reduces again at water tree tips for the larger deformation. At the same time, even though the yield strength drops by 22% from 60 °C to 80 °C, the effect of reduction of electric field force is more than that of reduction of yield strength for the water tree propagation. As a result, the water tree propagation will be slowed down from 60 °C to 80 °C.

## 5. Conclusions

This paper investigated the propagation characteristics of water trees at a wide temperature range and showed that the changing trends of water tree propagation are different at different temperature ranges. Water tree growth can be promoted at some temperature ranges, whereas water tree growth also can be suppressed at other temperature ranges. Based on the mutual effect between the electric field force and the mechanical properties of the material, the relationship between water tree propagation and temperature was explained. The conclusions are as follows:

At low-temperature region (below Tg), because of difficult movement of chains and slower diffusion of water molecules and ions, water tree propagation can be suppressed with the decrease in temperature. At normal temperature region (from 0–60 °C), water tree length presents the trend in the form of firstly increase and then decrease. Below 40 °C, due to volume expansion in water tree branches, the decrease of electric field force will inhibit the propagation of water trees. When the temperature is higher than 40 °C, because the further volume expansion can be limited by the free volume, the electric field force at water tree tips no longer decreases. However, the yield strength continues to reduce, and the growth of water trees can be promoted. At a high-temperature region (from 60–80 °C), because of the start in the movement of the crystalline phase, the deformation will greatly increase. As a result, the electric field force further reduces at water tree tips, and the water tree propagation will be suppressed again. Regardless of high temperature or low temperature, the water tree propagation is closely related to the mechanical properties of the material. With the increase in temperature, the increased deformation will reduce the Maxwell force at water tree tips and suppress the water tree propagation, whereas the decreased yield strength will promote water tree propagation. For this reason, the water tree propagation can be promoted or suppressed at different temperature ranges, which depends on who plays a dominant role.
